# A LAMP assay for the detection of *Bactrocera tryoni* Queensland fruit fly (Diptera: Tephritidae)

**DOI:** 10.1038/s41598-020-65715-5

**Published:** 2020-06-12

**Authors:** Mark J. Blacket, Arati Agarwal, Linda Zheng, J. Paul Cunningham, David Britton, Isarena Schneider, Brendan C. Rodoni

**Affiliations:** 1 Agriculture Victoria Research, AgriBio, 5 Ring Road, Bundoora, Victoria, 3083 Australia; 2 School of Applied Systems Biology, La Trobe University, Bundoora Victoria, 3083 Australia; 3 Department of Agriculture, Water and the Environment, Northern Australian Quarantine Strategy (NAQS), Cairns, Queensland 4870 Australia

**Keywords:** Molecular biology, Assay systems, Sequencing, Invasive species, Entomology

## Abstract

LAMP assays are targeted molecular tests for the rapid detection of species in the laboratory and field. We developed a LAMP assay for an economically important fruit fly species, Queensland fruit fly, *Bactrocera tryoni*. This assay was assessed against a broad panel of target and non-target species and found to be specific, only amplifying the target species and closest relatives, in a portable real-time fluorometer (Genie III) in under 15 minutes with an anneal derivative temperature of 82.5 ^o^C. The assay is sensitive to low levels of target DNA (>0.016 ng/µl), performing equally to the existing qPCR test. To enable retention of a physical voucher specimen, for potential morphological confirmation of LAMP results, a novel whole-specimen non-destructive DNA extraction method was developed, suitable for LAMP in the field. The stability of DNA extraction and LAMP reagents was tested under simulated and actual field conditions and shown to be robust. Our new assay now provides a portable molecular tool for the detection of this significant tephritid fruit fly pest species of biosecurity/quarantine concern. This has already proven invaluable for in-field diagnostics, providing real-time support influencing immediate actions, with negative results allowing the release of fruit produce, and positive results initiating fruit fly control measures.

## Introduction

Fruit flies belong to the large family Tephritidae, with more than 4000 described species worldwide^1,2^, with fewer than 10% of these species considered to be economic horticultural pests, while the majority, greater than 90% of species, are considered non-pests^2^. Many species are similar as adults and the larvae often cannot be identified to species using morphology^1,2^. There is a need to distinguish pest from non-pest species as adult fly traps use lures that are not species-specific^2^, and larvae found in fruit could be confused with fly larvae of other tephritid species or potentially other fly families, such as Drosophilidae and Lonchaeidae^3^. These limitations mean that there is a biosecurity and quarantine need for rapid and accurate molecular identification tools for the detection of pest species intercepted in the field, or at borders within and between nations^4,5^.

Existing molecular tools for the identification of morphologically similar species include both DNA barcoding^6,7^ and real-time qPCR for detection of specific pest fruit flies^8^. Neither of these approaches are currently easily applicable outside of molecular laboratories. LAMP, a DNA based molecular method using loop-mediated isothermal amplification^9^ provides a simple and portable molecular tool for very rapid (<1 hour) accurate in-field detection of specific target pest species of concern. Advantages of LAMP assays over other available molecular methods include the high specificity of LAMP, due to the use of six primer pairs designed from eight closely linked regions, rapid isothermal amplification, enhanced by the use of loop primers, as well as robustness and ease of use in the field^9,10^.

Globally there is a need for rapid, accurate identification of pest flies^1^. LAMP assays have previously been developed for specific target flies (Table 1), including Mediterranean fruit fly, *Ceratitis capitata*^11^ and several other high-priority fruit fly pest species within the genera *Bactrocera* and *Zeugodacus* that are commonly intercepted at national borders^5^. There is no published LAMP assay for one of the most serious polyphagous invasive fruit flies of economic and trade concern, the Australian endemic Queensland fruit fly *Bactrocera tryoni* (Froggatt) which occurs in eastern and northern Australia^12^ and has been introduced to several Pacific islands^2^. Queensland fruit fly is the most serious pest species in a closely related species group, referred to as the *B. tryoni* complex which includes four morphologically similar species, *B. tryoni, B. aquilonis, B. neohumeralis, B. melas*^13^, with the latter species often informally accepted as being synonymous with *B. tryoni*^13^. Currently, none of the *B. tryoni* complex species can be separated genetically from one another^4,14^.

**Table 1:** Published LAMP assays for identification of fly species.

Taxa	Group	Sample Type	DNA Extraction Method	TargetLocus	LAMP reagents	Amplification	Number of Primers	Visualisation	Melting temperature	Primer Targets	Reference
*Ceratitis capitata*	Mediterranean fruit fly	Adults, pupae, larvae, eggs	Spin column kit; QuickExtract; Chelex.	5.8 S rDNA (nuclear)	Custom made master mix	PCR Thermocycler	6	Fluorescence in tubes (SYBR Green)	Not tested	Singleplex	^11^
*Bactrocera dorsalis* group*, B. latifrons*, & *Zeugodacus cucurbitae*	Fruit flies	Larvae	Alkaline lysis solution (KOH)	5′-COI (mitochondrial)	Commercial master mix	Real-time fluorometer (Genie II), & Real-time qPCR thermocycler	6	Fluorescence measured in real-time fluorometer (SYBR Green)	Used for secondary confirmation of positive samples	Multiplex	^5^
*Dacus ciliatus*	Ethiopian fruit fly	Adults, pupae, larvae, eggs	Distilled water	5′-COI (mitochondrial)	Custom made master mix	PCR Thermocycler	4	Fluorescence in tubes (Calcein)	Not tested	Singleplex (nested PCR-LAMP)	^15^
*Drosophila suzukii*	Spotted winged drosophila	Adults	Spin column kit	Ds10_00012111 (nuclear)	Custom made master mix	PCR Thermocycler	4	Fluorescence in tubes (SYBR Green)	Not tested	Singleplex	^18^
*Anopholes gambiae* & *A. arabiensis*	Mosquitoes	Adults	Spin column kit; NaCl buffer.	IGS 28 S rDNA (nuclear)	Commercial master mix	PCR Thermocycler	4	Fluorescence in tubes (Calcein)	Not tested	Singleplex (species-specific assays, x 2)	^19^

In this study we report the use of a broad panel of target and non-target species of fruit fly and non-tephritid species to: (i) develop and test a LAMP assay for *Bactrocera tryoni* using a portable real-time fluorometer, Genie III, (ii) test the performance of the new LAMP assay compared with the existing species-specific real-time qPCR test and (iii) assess the performance of DNA extraction and LAMP reagents under typical field conditions.

## Results

### Specimens examined

All specimens examined in the current study (Table 2) were initially identified morphologically and confirmed through DNA barcoding. Cytochrome Oxidase I (COI) sequences of these specimens have been submitted to GenBank, accession numbers MT474870 - MT474916.

**Table 2:** Species assessed using the new *B. tryoni* LAMP assay.

Family	Tephritidae Tribe	Genus	Species*	n	Specimen source	Australian Species Distribution**	PHA (2018) Australian Pest Status***
Tephritidae	Dacini	*Bactrocera*	*B. aquilonis*	5	NAQS	WA, NT	Present, Pest
			*B. bancroftii*	2	NAQS	Qld, NSW	Present, non-pest
			*B. bryoniae*	2	NAQS	WA, NT, Qld, NSW	Present, Pest
			*B. cacuminata*	2	VAIC	Qld, NSW, Vic	Present, non-pest
			*B. dorsalis*	4	VAIC	Absent, exotic pest	Exotic, Pest
			*B. endiandrae*	4	NAQS	Qld, NSW	Present, non-pest
			*B. erubescentis*	2	NAQS	Qld	(no known hosts)
			*B. frauenfeldi*	2	NAQS	Qld	Present, Pest
			*B. jarvisi*	2	VAIC	WA, NT, Qld, NSW	Present, Pest
			*B. manskii*	2	NAQS	Qld	(very few hosts)
			*B. musae*	2	NAQS	Qld	Present, Pest
			*B. neohumeralis*	2	NAQS	Qld, NSW	Present, Pest
			*B. tryoni*	2	VAIC	NT, Qld, NSW, Vic	Present, Pest
		*Dacus*	*D. newmani*	2	VAIC	WA, NT, Qld, NSW, SA, Vic	(milkweed host)
		*Zeugodacus*	*Z. cucumis*	2	VAIC	NT, Qld, NSW	Present, Pest
			*Z. cucurbitae*	2	NAQS	Absent, exotic pest	Exotic, Pest
			*Z. strigifinis*	2	NAQS	Qld	(no known hosts)
			*Z. fallacis*	2	NAQS	Qld	(no known hosts)
	Ceratitidini	*Ceratitella*	*C. loranthi*	2	VAIC	WA, NT, NSW, SA, Vic	(mistletoe host)
		*Ceratitis*	*C. capitata*	4	VAIC	Exotic, present only in WA	Present, Pest
	Acanthonevrini	*Dirioxa*	*D. pornia*	2	VAIC	WA, Qld, NSW, SA, Vic	Present, non-pest
Lonchaeidae	N/A	*Lamprolonchaea*	*L. brouniana*	2	VAIC	WA, NT, Qld, NSW, SA, Vic	N/A
Drosophilidae	N/A	*Drosophila*	*D. melanogaster*	2	VAIC	WA, NT, Qld, NSW, SA, Vic, Tas	N/A

*Adult specimens, except *C. capitata* and *Z. cucumis*. **Distribution of Tephritidae from^20^, Lonchaeidae from^3^, Drosophilidae from ALA (https://www.ala.org.au/), abbreviations refer to Australian State names. ***Plant host information in brackets from^21^, for species not included in^2^. Abbreviations: VAIC, Victorian Agricultural Insect Collection; NAQS, Northern Australian Quarantine Strategy.

### Development of the LAMP assay

**Figure 1:**
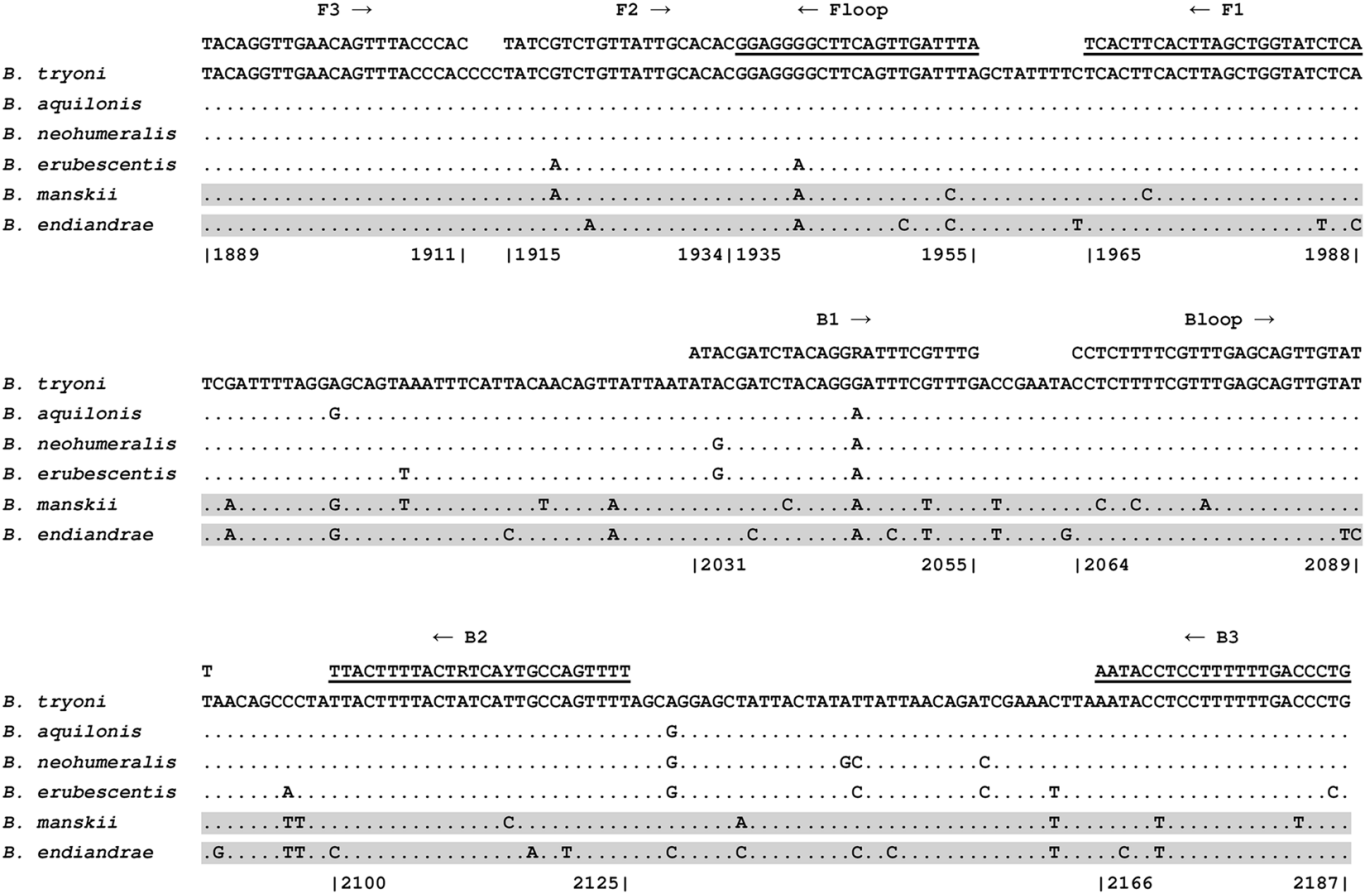
*B. tryoni* LAMP assay primer alignment, showing target species and their closest relatives tested. Eight primer regions have been used for designing six primer pairs. Reverse primers are underlined; FIP (5′-3′) is made by combining F1 (reverse compliment) and F2; BIP (5′-3′) is made by combining B1 (reverse compliment) and B2. Numbers below alignment refer to base pair positions (1889 to 2187) in the *B. tryoni* mitochondrial genome^22^, GenBank accession NC_014611. DNA sequences shown here were generated in the current study. Grey shading indicates genetically similar species that did not amplify, or amplified very late (>24 min.).

Novel LAMP primers (Table 3) were developed to target the *B. tryoni* complex for the 5′ region of the mitochondrial COI locus (Fig. 1). This is the standard DNA barcoding gene, and has been employed in other recent fruit fly LAMP assays^5,15^. This region is highly variable, with enough resolution to differentiate between most insect species and consequently, has been sequenced from more fruit fly species than any other locus^4^. The *B. tryoni* LAMP assay consists of six primers, the outer forward primer F3, the outer backward primer B3, the inner forward primer FIP, the inner backward primer BIP, the forward loop primer Floop and the backward loop primer Bloop (Fig. 1). We assessed the primer ratio (F3/B3: FIP/BIP: Floop/Bloop) for the assay and found the optimal ratio to be 1:6:3, with the final concentrations of 0.4 µM, 2.4 µM and 1.2 µM for the F3/B3, FIP/BIP and Floop/Bloop primers respectively. The use of loop primers in this assay was found to result in more rapid amplification times.

**Table 3:** LAMP primer sequences and parameters.

Primers	Sequence 5′-3′	Primer length (bp)	Predicted Tm, annealing temperature (°C)	Degeneracy of primer
Btryoni_F3	TACAGGTTGAACAGTTTACCCAC	23	60.3	None
Btryoni_B3	CAGGGTCAAAAAAGGAGGTATT	22	57.7	None
Btryoni_FIP	TGAGATACCAGCTAAGTGAAGTGATATCGTCTGTTATTGCACAC	44	71.4	None
Btryoni_BIP	ATACGATCTACAGGRATTTCGTTTGAAAACTGGCARTGAYAGTAAAAGTAA	51	70.4	8
Btryoni_FL	TAAATCAACTGAAGCCCCTCC	21	60.3	None
Btryoni_BL	CCTCTTTTCGTTTGAGCAGTTGTATT	26	59.8	None

The F2 and B2 primer regions of FIP and BIP are underlined.

### Performance of the LAMP assay

**Figure 2:**
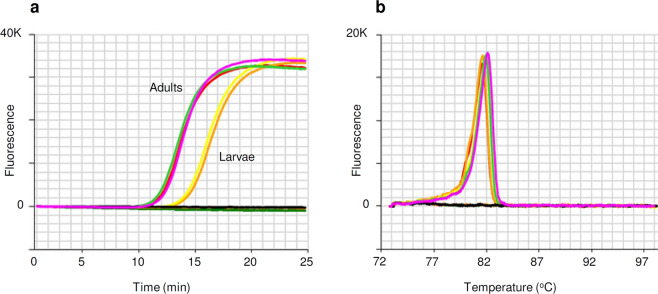
Optimised LAMP assay performed on *B. tryoni* adults and larvae. (**a**) Amplification profile, with positive samples amplifying in <15 minutes. (**b**) Anneal derivative of LAMP amplicons, with an anneal derivative of 82.5 °C.

**Figure 3:**
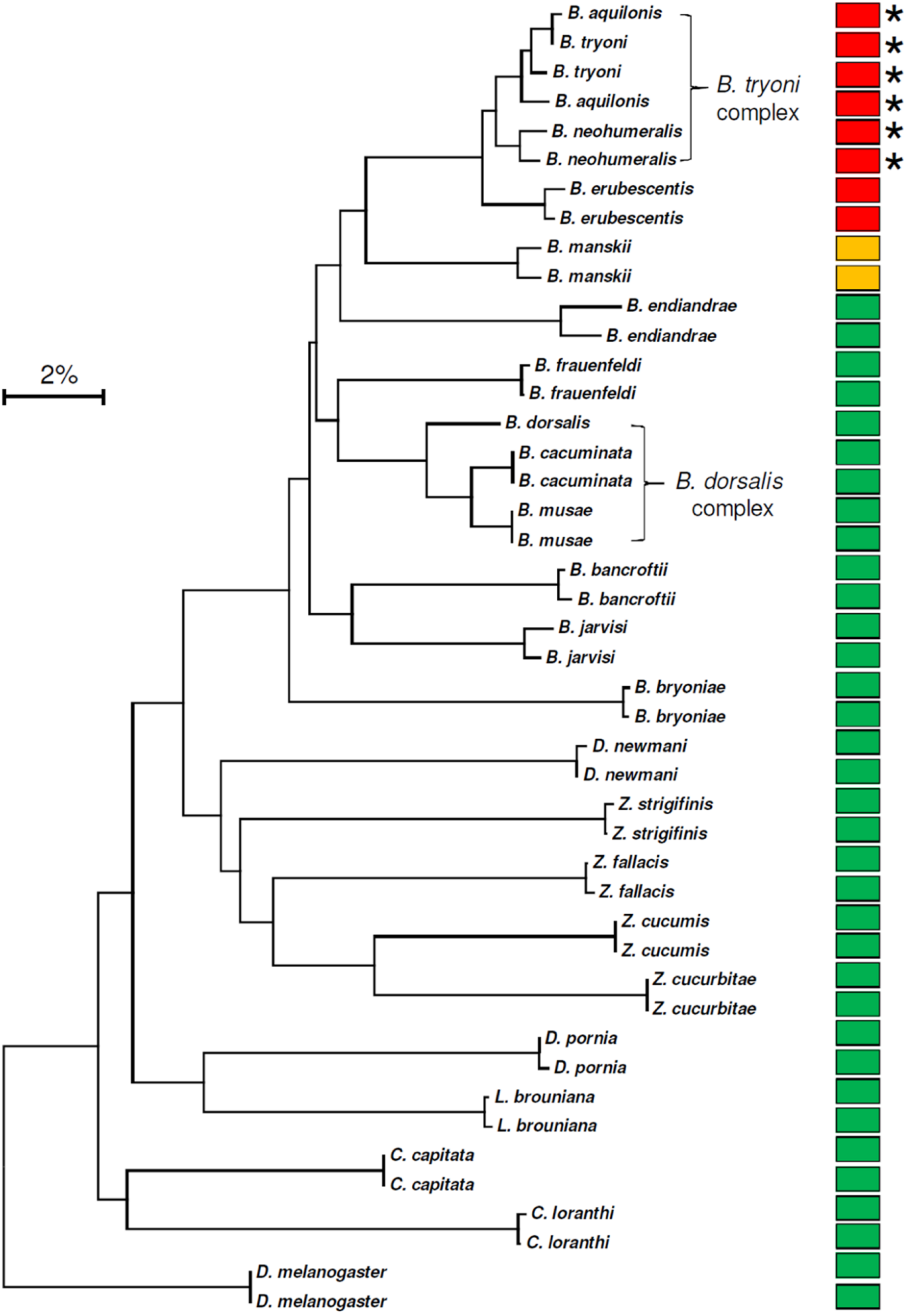
Neighbour joining tree, COI DNA sequences. The *B. tryoni* LAMP assay targets (species complex) are indicated by an asterix, red indicates positive amplification (approx. <15 min.), orange indicates very late amplification (>24 min.), green indicates no amplification.

The *B. tryoni* LAMP assay usually produces amplification of the target species in less than 15 minutes and an anneal derivative temperature of approximately 82.5 °C (Fig. 2), with amplification in under 25 minutes considered positive. The assay was found to work across the *B. tryoni* complex, detecting *B. tryoni, B. aquilonis* and *B. neohumeralis*, as well as the genetically similar *B. erubescentis*, which is only approximately 2% divergent (Fig. 3). The next most similar species tested, *B. manskii*, approximately 5% divergent (Fig. 3), produced very late LAMP amplification time at >24 minutes, with an anneal derivative temperature of 82.3 °C. All other non-target species in Table 2 produced negative LAMP results.

**Figure 4:**
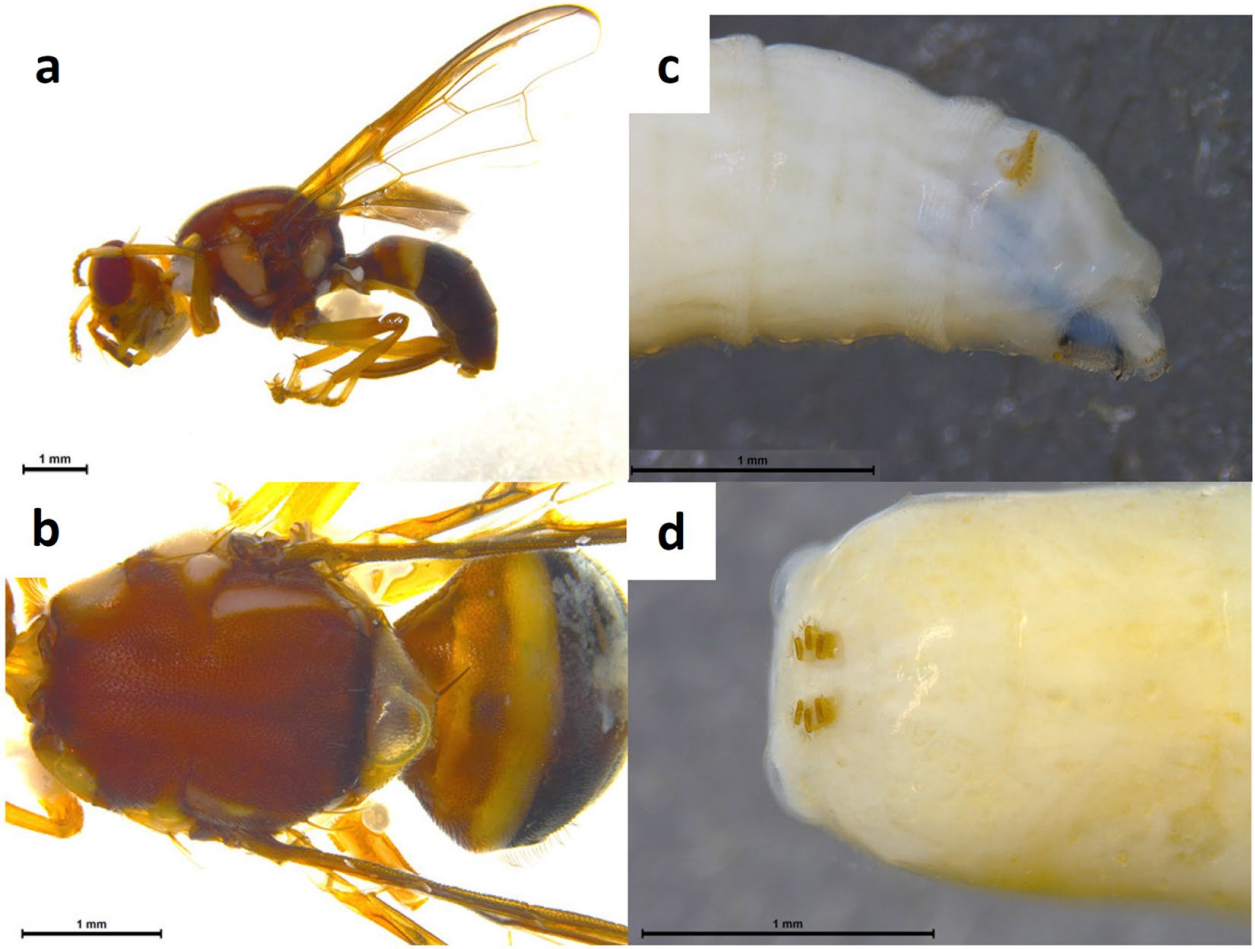
*B. tryoni* adult (left) and larva (right) after DNA extraction using QuickExtract. (**a**) adult lateral, (**b**) adult dorsal, (**c**) larva anterior, (**d**) larva posterior.

A novel whole-specimen non-destructive DNA extraction method using QuickExtract, was developed for obtaining *B. tryoni* DNA from adult, larva, pupa and egg, suitable for the LAMP assay. We tested this “crude” DNA extraction method to both enable DNA extraction in the field, using the LAMP machine as a heating block, and to allow retention of complete morphological voucher specimens for further examination (Fig. 4) if required for morphological or molecular verification of species identification. All life stages of *B. tryoni* samples generated positive amplification curves within 15 minutes (Table 4a).

**Table 4:** Performance of the LAMP assay.

Samples	n	Amplification Time (minutes)			Anneal Derivative Temperature (^o^C)		
		Average	SD	Min-Max	Average	SD	Min-Max
***a***							
Adult	6	12.6	0.9	12.0–14.0	81.8	0.2	81.4–82.0
Larva	2	11.5			82.5		
Pupa	2	14.7			82.5		
1 Egg	2	9.5			82.2		
2 Eggs	2	8.0			81.9		
5 Eggs	2	7.4			81.9		
***b***							
*B. tryoni* larva	1	11.3			82.2		
*B. tryoni* larva + 5 *Drosophila* larvae	1	12.3			82.3		
*B. tryoni* larva + 10 *Drosophila* larvae	1	12.8			81.7		
*B. tryoni* larva + 5 *Drosophila* adult	1	12.3			82.0		
***c***							
*B. tryoni* adult	18	11.9	1.8	9.5–17.2	82.2	0.2	81.6–82.6
***d***							
*B. tryoni* adult	18	12.3	1.4	10.0–14.2	81.9	0.3	81.4–82.4
*B. tryoni* larva	18	15.0	1.4	13.0–18.0	82.0	0.2	81.7–82.3
*B.tryoni* (positive control)	11	12.0	0.6	11.0–12.5	82.3	0.2	81.9–82.6
*Drosophila* larva	26	Nil	Nil	Nil	Nil	Nil	Nil

**a)** QuickExtract DNA obtained from all *B. tryoni* lifestages. **b)** Samples containing multiple specimens of mixed species, *B. tryoni* and *Drosophila*. **c)** Preserved adult flies from traps. **d)** Blind panel testing, *B. tryoni* and *Drosophila* (workshop results).

Crude extractions proved robust, even when multiple non-target *Drosophila* larvae were included in mixed species samples (Table 4b). The average amplification time with five to ten *Drosophila* larvae or adults pooled with a single *B. tryoni* larva was 12.5 minutes.

Crude DNA from eighteen adult *B. tryoni* specimens (trap samples) stored for more than six-months at room temperature (approx. 20 °C) in absolute ethanol was also extracted as above, generating positive amplification from all samples (Table 4c).

The in-field applicability of the *B. tryoni* LAMP assay was tested by previously untrained research personnel participating in a short workshop training session, extracting “crude” DNA from *B. tryoni* and *Drosophila* adults and larvae. The workshop participants produced the expected LAMP results from the (blind panel) unidentified specimens (Table 4d).

**Figure 5:**
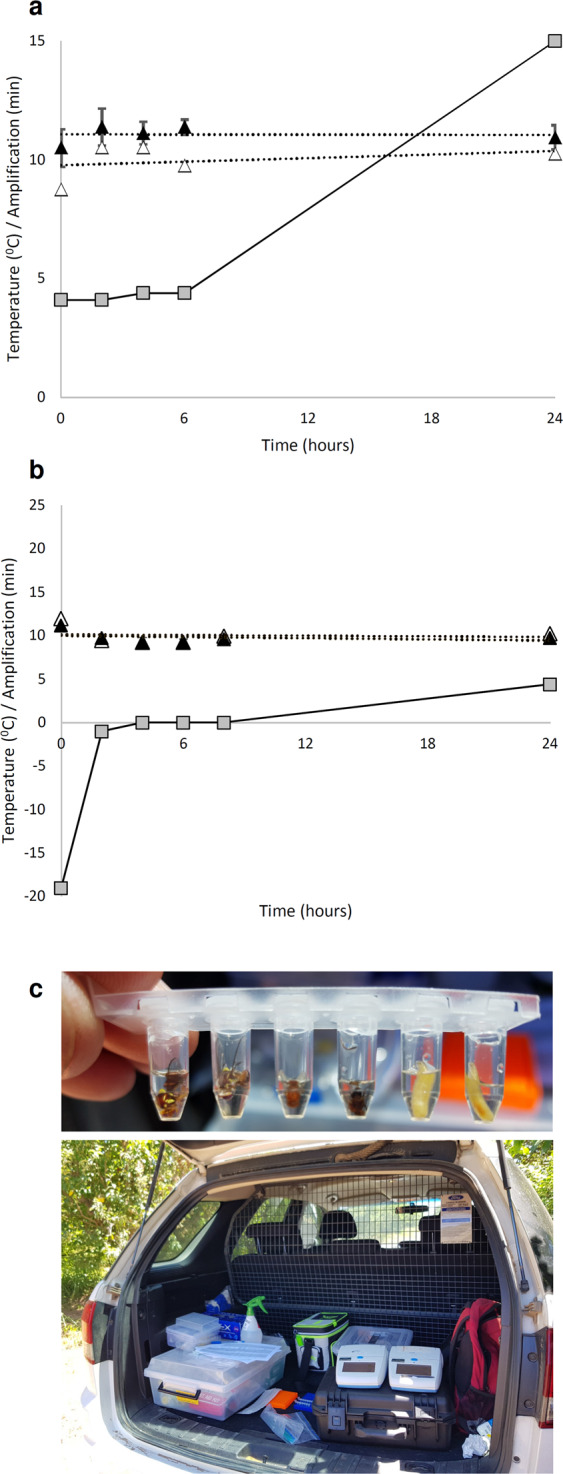
Evaluation of field conditions. (**a**) QuickExtract reagent temperature/time stability “crude” DNA extractions. (**b**) *B. tryoni* LAMP master mix temperature/time stability. Temperature, grey squares & solid line; *B. tryoni* LAMP assay, “crude” extracts, triangles; positive control samples, “clean” DNA extracts, unfilled triangles; error bars standard deviations; dotted lines linear regressions. (**c**) *B. tryoni* DNA extraction (upper) and LAMP assay (lower) being conducted in the field.

The DNA extraction buffer and pre-prepared LAMP master mixes were further tested under typical field conditions, i.e. with reagents kept on ice for 24 hours. Under these simulated in-field conditions, the extraction buffer performed consistently over a day (24 hours), with the *B. tryoni* crude extractions consistently amplifying in under 12 minutes, despite the reagent temperature rising (Fig. 5a). Similarly, no delays in amplification times or off-target amplification were observed for the pre-prepared *B. tryoni* LAMP master mix (Fig. 5b). Adult flies from traps and larvae collected from fruit also tested positive in actual field conditions (Fig. 5c).

### Sensitivity of LAMP and qPCR assays

**Figure 6:**
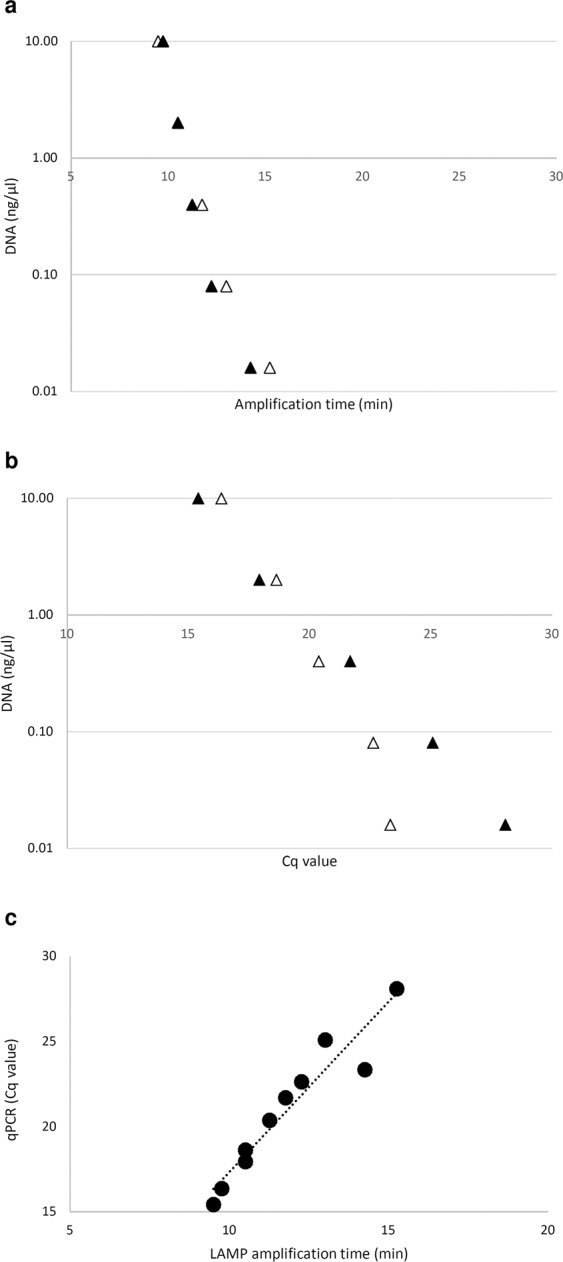
Comparison of *B. tryoni* LAMP and *B. tryoni* real-time qPCR assays. (**a**) LAMP, DNA dilution series amplification times for two biological replicates of *B. tryoni* (triangles). (**b**) Real-time qPCR, DNA dilution series Cq values, DNA samples as above. (**c**) Direct comparison of new LAMP assays and qPCR assays on dilution series DNA, symbols represent average replicates of *B. tryoni* (circles), linear regression R^2^ = 0.91.

The sensitivity of the *B. tryoni* LAMP assay was compared with the existing laboratory-based *B. tryoni* (species complex) qPCR test, following published protocols^8^. We found that the *B. tryoni* qPCR test produced reliable amplification using the published *C. capitata*^8^, rather than *B. tryoni*[8], conditions using our laboratory reagents. Both LAMP and qPCR performed similarly on serial DNA dilutions down to 1:625, with positive amplification from high to very low DNA concentrations (Fig. 6a,b). At the lowest DNA concentrations, of 0.016 ng/µl, the average LAMP amplification time was <15 minutes, compared with an average Cq value of 26 using qPCR (Fig. 6a,b), with greater variation observed between qPCR than LAMP on the same biological replicates at lower DNA concentrations (Fig. 6a,b). A strong relationship between LAMP amplification times and qPCR Cq values from the same dilution samples was observed (R^2^ value 0.91, Fig. 6c). As in qPCR, amplification in the LAMP assay was found to become slower in a predictable manner as DNA template concentrations reduced, showing a strong relationship between increased amplification times and decreasing DNA concentrations (R^2^ values 0.88 & 0.93, Fig. 6a,b respectively).

## Discussion

In this study a new Queensland fruit fly LAMP assay was developed and tested. We characterised COI sequence variation from a wide range of fruit flies and tested these species with the novel *B. tryoni* LAMP assay. The target fruit fly, *B. tryoni*, belongs to a species complex, where the COI locus cannot distinguish close relatives^4,14^, and these species also amplified with the assay, as did some other very closely related non-pest species. The two Australian endemic species, *B. erubescentis* and *B. manskii*, which also amplified have geographic ranges that are relatively restricted to north-eastern Australia and neither is known to utilise horticultural fruit as hosts (Table 2, Hancock *et al*. 2000). It is likely that some other species which are very similar to *B. tryoni*, such as *B. curvipennis* and *B. humilis*^4^ which have not been tested here due to unavailability of DNA samples, would also amplify using the *B. tryoni* LAMP assay.

Multiple DNA extraction methods have been used in previous published fly LAMP assays, including various commercial kits and crude extraction methods (Table 1). In the current study both kit-based and crude DNA extraction approaches were tested, with both found to be suitable for *B. tryoni* LAMP. An advantage of the crude in-field DNA extraction method used here (extracting DNA from intact specimens using QuickExtract) is the retention of a physical voucher specimen. Intact voucher specimens provide specimen-based evidence for the presence/absence of the target species and could be used for morphological species confirmation, if required.

LAMP performed well compared with qPCR, proving to be sensitive even at low DNA concentrations of *B. tryoni* DNA. LAMP was found to be effective on all fruit fly life stages, including eggs and larvae, the stages most likely to be intercepted in host fruit. Testing mixed samples which included both target (*B. tryoni*) and non-target (*Drosophila*) samples also proved robust. Advantages of the *B. tryoni* LAMP assay over existing qPCR tests include ease of in-field use and quicker running times. Previously published fly LAMP protocols (Table 1) have mostly used standard PCR thermocyclers, rather than specialised equipment for LAMP. The portable real-time fluorometer used here allowed the amplification time to be accurately determined and recorded, as well as providing confirmation that the correct target DNA region had been amplified, through the target anneal derivative profiles.

Testing of the assay under simulated field conditions showed that both the DNA extraction solution and the pre-prepared assay reagents were stable on ice, despite the temperature slowly rising over the day. Overall, crude in-field DNA extractions were marginally slower to amplify than clean (positive control) DNA extracts. The field protocols developed here were successfully used in the field and for training workshops involving large numbers of personnel who had never previously conducted LAMP. Specimen preservation was not extensively tested here. However, it is likely that field collected samples (i.e. trapped adults, intercepted larvae) may start to degrade through environmental stresses such as heat and humidity, unless specimens are collected in appropriate preservatives^16^. This is an area requiring further study.

Our assay provides a new portable molecular tool which can now be used in the laboratory or in the field for the detection of a significant tephritid fruit fly species of biosecurity and quarantine concern. The *B. tryoni* LAMP test has already been adopted on a trial basis for surveillance in Victoria, Australia. In the 2018/2019 fruit fly season it proved to be invaluable for in-field diagnostics, with two (one larva, one adult) out of thirteen samples testing positive. Since this assay takes less than an hour, the technology provides real-time support that can influence immediate actions, allowing fruit produce to be released for shipping after negative results (maintaining area freedom), and initiating fruit fly treatment measures following positive results. The applicability of this method for surveillance is further evident by the assay already being taken up by several other fruit fly surveillance programs in plant health laboratories in Australia. The addition of other LAMP assays for pest fruit flies in the future, will no doubt prove even more valuable for detecting and differentiating tephritid species.

## Materials and Methods

### Specimens examined

Twenty-three species of flies were assessed in this study. Specimens were obtained from the AgVic Victorian Agricultural Insect Collection (VAIC) or from recently trapped flies collected from northern Australia, through the Department of Agriculture, Water and the Environment (DAWE) Northern Australian Quarantine Strategy (NAQS) program. Species were initially identified morphologically using published literature^1,2,17^. The specimens examined, together with their known Australian distributions and pest status, are listed in Table 2. The species were chosen to include examples of the target species and the closest known relatives for testing the specificity of our novel LAMP assay, as well as a range of other Australian Tephritidae, Drosophilidae and Lonchaeidae pest and non-pest flies (Table 2).

### DNA extractions

DNA was extracted from dry or ethanol (absolute) preserved adult whole specimens, larva, heads or legs of adults using DNeasy Blood and Tissue extraction kit (Qiagen), following the manufacturers protocol (Table 2). These samples provided “clean” DNA preparations to use for DNA barcoding for species identification and as positive controls for testing the LAMP assay. All specimens were identified morphologically, prior to DNA extraction. Species identification of all specimens (Table 2) was also confirmed using standard DNA barcoding methods^4^.

Additional “crude” DNA extractions were prepared from whole specimens of *B. tryoni* (eggs, larvae, pupae and adults) using QuickExtract DNA extraction solution 1.0 (Epicentre, USA). Fifty microliters of QuickExtract DNA extraction solution was pipetted into each 8 well strip of LAMP PCR tubes (OptiGene, UK). Fly samples were removed from ethanol and air dried on a paper towel for approx.1 min. Single whole adult fly (placed head down), larva, pupa, single and/or multiple eggs were pricked with an entomology pin (single use to prevent cross contamination), which was used both to help release DNA from specimens and transfer each specimen into a well. Each sample was immersed in QuickExtract DNA extraction solution with up to six samples processed simultaneously in the portable real-time fluorometer (Genie III, OptiGene, UK). The protocol used the Genie III as an incubator at 65 °C for 6 min followed by 98 °C for 2 min (Total run time = 8 min), following the QuickExtract manufacturers recommendations. The DNA was quantified using a NanoDrop ND-1000 Spectrophotometer (Thermo Fisher, Australia) and stored at −20 °C. Whole specimens post QuickExtract DNA extraction were retained and visually examined to confirm morphological identification features were still present.

### Development and evaluation of LAMP assay

#### LAMP primer design

We developed new LAMP primers in this study to target the *B. tryoni* complex, including *B. tryoni, B. neohumeralis and B. aquilonis*. This assay was designed to target the 5′ region of the mitochondrial COI locus. All available sequences of the COI coding region of the target species were retrieved from the BOLD Systems public database and used for the LAMP primer design. A total of 54, 4 and 24 sequences were available for *B. tryoni, B. neohumeralis* and *B. aquilonis* respectively as of 11^th^ September 2017. Alignments generated from these sequences are referred to as the “target” set. Reference fruit fly DNA sequences from the *Bactrocera* genus other than the target species of the *B. tryoni* complex were also retrieved from the BOLD Systems database (2922 sequences as of 11^th^ September 2017) and used for comparison. The alignment generated from these non-target sequences is referred to as the “non-target” set. The sequences were aligned using the alignment program Clustal Omega provided at The European Bioinformatics Institute (EMBL-EBI; https://www.ebi.ac.uk/Tools/msa/clustalo/) using default parameters. Highly conserved regions within the target sequence alignments were identified manually, assessed for their suitability as LAMP primers and chosen based on the following criteria: i) sites with least degree of degeneracy within the target species yet still sufficiently different (preferably > 3 bases different) to the same region in the non-target sequence alignment; ii) sites with the least degree of self-annealing and loops formation, especially at the 3′ end and iii) their relative positions to each other in accordance with the design guidelines stated by the Primer Explorer software (http://primerexplorer.jp/e/). Primer degeneracy was added to account for known *B. tryoni* complex sequence variation based on the target alignment.

#### LAMP primer ratio optimisation

A primer master mix was prepared first for the assay for ease of reaction setup. The primer master mix was prepared by adding the specified amount of each of the six primers (Table 3) as per the ratio requirement for outer forward primer (F3), outer backward primer (B3), forward inner primer (FIP), backward inner primer (BIP), forward loop primer (Floop) and backward loop primer (Bloop). Due to the higher concentration required for the inner and loop primers, both were used at a concentration of 100 µM whereas the outer primers were diluted to a working concentration of 10 µM for ease of calculation. A 100 µL volume of primer master mix 1:6:3 (F3/B3: FIP/BIP: Floop/Bloop) was prepared by adding 10 µL each of F3/B3, 6 µL each of FIP/BIP, 3 µL each of Floop/Bloop and 62 µL of Ultrapure water (Invitrogen, Australia). The final concentrations of each LAMP primer within the newly developed assay were determined empirically based on published literature and previous work with LAMP (not published). The concentrations for the outer primers F3 and B3 were tested at 0.2 µM (5 µL of primer master mix and 5 µL of H_2_O), 0.3 µM (7.5 µL of primer master mix and 2.5 µL of H_2_O) and 0.4 µM (10 µL of primer master mix only) with the following ratios 1:6:3, 1:8:4, 1:10:5 and 1:12:6 for outer primers: inner primers: loop primers, for the *B. tryoni* LAMP assay. Optimisation of primer ratio is critical in the development of the LAMP assay, firstly to determine the optimum amplification time of target species (within 25 minutes), and secondly to be able to achieve a consistent annealing temperature for all the positive DNA samples. Negative samples and non-target species should not amplify and the anneal derivative should remain as a flat line.

#### LAMP assay conditions

Each LAMP reaction was conducted in a total volume of 25 µl containing 14 µl of Isothermal Master Mix (Iso-001, OptiGene, UK), 10 µl of the primer master mix containing all 6 primers at the appropriate concentrations and 1 µl of template DNA. A one microliter disposable plastic loop was used to add “crude” QuickExtract DNA, ensuring that a fresh loop was used for each sample. The use of disposable plastic loops eliminates the need for pipettes when LAMP is used outside a laboratory, a necessity for effective field deployment.

“Clean” DNA extracted from the target species using the DNeasy Blood and Tissue extraction kit was used as positive controls for the LAMP assay. A no template control (NTC) was included in each test to detect reagent contamination. All the LAMP assays were run in the Genie III at 65 °C for 25 min followed by an annealing curve analysis from 98 °C to 73 °C with ramping at 0.05 °C/s. The total run time is approximately 35 minutes.

Once the run has finished, the amplification and anneal derivative curves can be visualised on the Genie III screen to ensure that amplification has occurred as expected. Non-target species and NTCs are expected to have flat amplification lines. The time of amplification (minutes) and anneal derivative temperature (°C) are recorded from the Results Table displayed by the Genie III. The amplification time and the anneal derivative temperature of all the samples are compared against the positive control/s to confirm that no false-positive or false-negative has amplified.

The date, Genie III serial number and the run number of each LAMP assay completed on the machine was recorded so that the run files could be transferred and analysed using a PC version of the software Genie Explorer version V2.0.6.3. LAMP assays in this study were visualised in the blue channel of the Genie III.

#### Analytical sensitivity of the LAMP assay compared with qPCR

A five-fold serial dilution of a “clean” DNA extract was prepared using ultrapure water from two biological replicates of *B. tryoni*. Starting DNA concentrations were quantified using a NanoDrop ND-1000 Spectrophotometer (Thermo Fisher, Australia). *B. tryoni* DNA was serially diluted from 10 ng/µl to 0.016 ng/µl (1:1 to 1:625). Sensitivity of the LAMP assay was tested using the serially diluted DNA in the Genie III, following the same assay conditions as mentioned above. The time of amplification and anneal derivative temperatures were recorded for all samples.

The same serial dilution of DNA extracts was also used in real-time qPCR assay. The primers (manufactured by Sigma), probes (manufactured by Applied Biosystems and Sigma) and cycling conditions used were as published for Medfly^8^, including lowering the annealing temperature to 58 °C. Real-time qPCR was performed in a Rotor-Gene Q (Qiagen, Australia) in a total volume of 25 µl. Each reaction mixture included 12.5 µl Platinum Quantitative PCR SuperMix-UDG (Invitrogen, Australia), 0.5 µM of each forward and reverse primers, 0.2 µM Taqman probe, 5 µl of template DNA and made up to 25 µl with RNA-free water. An NTC with 5 µl of water instead of DNA was included in each run to check for reagent contamination. The thermal cycling conditions consisted of a two-step denaturation: 2 min at 50 °C and 10 min at 95 °C, followed by 35 cycles of amplification in a two-step procedure: 95 °C for 10 seconds and 58 °C for 1 min. The Cq value (cycling quantification value) of the 5 dilutions for the two biological replicates of the target species were recorded for comparison with the time of amplification obtained from the LAMP assays.

## Data Availability

GenBank, accession numbers MT474870 - MT474916.
